# The liver X receptor agonist LXR 623 restricts flavivirus replication

**DOI:** 10.1080/22221751.2021.1947749

**Published:** 2021-07-05

**Authors:** Luwanika Mlera, Danielle K. Offerdahl, David W. Dorward, Aaron Carmody, Abhilash I. Chiramel, Sonja M. Best, Marshall E. Bloom

**Affiliations:** a Biology of Vector-Borne Viruses Section, Laboratory of Virology, NIAID/NIH, Hamilton, MT, USA; b Microscopy Unit, Research Technologies Branch, NIAID/NIH, Hamilton, MT, USA; c Research Technologies Branch, NIAID/NIH, Hamilton, MT, USA; d Innate Immunity and Pathogenesis Section, Laboratory of Virology, NIAID/NIH, Hamilton, MT, USA

**Keywords:** Flavivirus, Zika virus, Powassan virus, liver X receptor, LXR 623, virus restriction

## Abstract

The vector-borne flaviviruses (VBFVs) are well known for causing great misery and death in humans worldwide. The VBFVs include those transmitted by mosquitos, such as Zika virus (ZIKV), dengue virus; and those transmitted by ticks including the tick-borne flavivirus serocomplex and Powassan virus (POWV). Two of our recent reports showed that intracranial POWV infection in the reservoir host, *Peromyscus leucopus*, was restricted and caused no overt clinical disease. Several modes of analyses suggested activation of the LXR pathway. Activation of the LXR pathway leads to increased efflux of cholesterol from cells and consequent disturbances in membrane biogenesis. Because VBFV replication is dependent on membrane biogenesis, we evaluated the effect of an LXR agonist (LXR623) on POWV and ZIKV infection and observed that the compound impaired permissive replication of both viruses in a human neuroblastoma SK-N-SH cell line. The LXR agonist resulted in failure of the viruses to induce ER expansion and elaborate vesicle formation, suggesting that the efflux of cholesterol was part of the antiviral mechanism. We also observed that the LXR agonist contributed to the mechanism of virus suppression by increased expression of mRNAs encoding for the antiviral cytokines CXCL10, RANTES and IFN1β. In sharp contrast, a LXR antagonist (GSK2033) had no significant effect on VBFV replication. We conclude that LXR623 impairs flavivirus replication by stimulating cellular antiviral factors.

## Introduction

The vector-borne flaviviruses (VBFVs) are a notorious group of agents capable of causing severe and life-threatening disease in humans and animals. VBFVs are vectored by either mosquitoes (MBFVs) or ticks (TBFVs). The better known MBFVs include dengue virus (DENV), yellow fever virus (YFV), West Nile virus (WNV) and Zika virus (ZIKV). Together, these viruses infect more than 100 million people annually, and the symptoms can include febrile illnesses, hemorrhages and shock (DENV), hepatic disease (YFV) and incapacitating encephalitis (WNV) [1–3]. ZIKV recently emerged in an outbreak of epidemic proportions in Latin American countries with consequences that included microcephaly in neonates who contracted the infection *in utero* [4]. Additional complications of ZIKV are varied and include Guillian Barre syndrome in adults [5].

Prominent TBFVs belong to the tick-borne encephalitis virus (TBEV) serocomplex, and include Powassan virus (POWV), the only TBFV known to circulate in North America. TBEV serotypes are quite common in European countries where they cause more than 15,000 cases annually despite the availability of effective vaccines [6]. POWV is considered a reemerging virus and there has been a distinct and troublesome increase in the number of cases in eastern and north eastern USA [7]. Although it is well-appreciated that the VBFVs are a major public health problem, there are few vaccines for these viruses and licensed treatments are still elusive.

An understanding of the precise cellular metabolic pathways enabling VBFV infection may aid in development of effective countermeasures. Several reports have shown that flavivirus replication is dependent on manipulation of lipid metabolism and membrane biogenesis [8–10]. We and others showed massive expansion and rearrangements of membranes on which flavivirus replication occurs are derived from the endoplasmic reticulum (ER) [9, 11, 12]. In addition, flavivirus replication is also characterized by the generation of vesicular replication compartments derived from ER lamellae [9, 11, 13, 14]. The generation of these membrane-bound vesicles and expansion of the ER stress lipid metabolism in infected cells [15]. Indeed, several reports have indicated that perturbation of lipid metabolism can negatively affect flavivirus replication [16, 17].

We recently studied transcriptomic changes in the brain of *Peromyscus leucopus* mice following POWV infection. The results indicated activation of the liver X receptor (LXR/RXR) pathway following intracranial challenge with virus [18], a result that would likely lead to enhanced efflux of cholesterol from cells. The liver X receptors are 2 nuclear receptors (LXR-α and LXR-β) that are integral to regulating lipid metabolism and inflammatory signaling [19]. Oxysterols were initially identified as the ligands for LXR receptors, and it was observed that mice lacking LXR-α had massive accumulations of cholesterol when fed high-cholesterol diets [20]. In our report, we hypothesized that the mild inflammatory changes observed and lack of severe pathology in this reservoir species resulted partly from activation of the LXR pathway [18].

In this current study, we explored the impact of perturbing the LXR pathway using the LXR agonist LXR 623 (WAY252623) on Zika and Powassan virus replication in a relevant permissive cell line. Our results demonstrate that activation of LXR by LXR 623 negatively impacts flavivirus replication, and that this is accompanied by disruption of flavivirus-induced vesicle biogenesis and upregulation of antiviral cytokine production. These results point to a role for non-interferon mediated antiviral responses in the cellular control of VBFV infections and at the possible utility of developing LXR agonists as flavivirus antivirals.

## Materials and methods

### Cells and viruses

The human neuroblastoma cell line SK-N-SH (ATCC) was maintained in EMEM containing 10% FBS (ATCC) without antibiotics.

The Ugandan Zika virus (strain MR766) and the Brazilian Paraiba strain were kind gifts from Dr. Stephen Whitehead (NIAID/NIH). The Paraiba strain is a clinical isolate from a patient and was isolated by Dr. Pedro F.C Vasconcelos, Instituto Evandro Chagas, Brazil. Powassan virus (LB strain) was a kind gift from Dr. Robert Tesh. The viruses were propagated in Vero cells (ATCC) and titers were determined by plaque assay (ZIKV) or immunofocus assay (POWV) on Vero cells.

### Evaluation of the effect of LXR-β agonists on flavivirus replication

SK-N-SH cells were seeded at 5 × 10^5^ cells per well in triplicate on a 6-well plate. The cells incubated overnight in normal EMEM, EMEM containing DMSO, EMEM containing either 50 µM or 100 µM of the LXR-β agonist LXR 623 (Sigma-Aldrich). In different wells, cells were incubated with the LXR-β antagonist GSK2033 at 50 µM or 100 µM. LXR 623 and GSK 2033 were dissolved in DMSO to 100 mM stocks.

The next day, cells were infected with either ZIKV MR766, ZIKV Paraiba, or POWV LB at a multiplicity of infection of 0.01 at 37°C with rocking. The infecting inoculum was removed followed by washing the cells with PBS twice and addition of complete EMEM or EMEM containing DMSO, 50 or 100 µM LXR 623, or EMEM containing either 50 or 100 µM GSK2033. Aliquots were removed from the infected cultures and stored at −80°C until virus titration.

Virus titrations were performed by serial dilution of samples in complete DMEM (containing 10% FBS; Gibco) and Anti–Anti (Gibco). The diluted samples were plated in duplicate onto confluent Vero cells and adsorption for 1 h at 37°C with rocking. The infecting inoculum was aspirated followed by washing the cells with PBS 2 times. Cells were overlaid with DMEM containing 1% carboxymethylcellulose and incubated for 5 days. The overlay was aspirated, and cells were fixed in 10% formalin for 1 h, followed by washing in water. The monolayers were stained with crystal violet for 10 min and the stain was washed off with water to visualize and enumerate plaques.

Powassan virus titers were determined by an immunofocus assay as described before [11].

### Cell proliferation assay

SK-N-SH cells were seeded in 96-well plates at 10^4^ cells/well and incubated overnight. The next day, EMEM was aspirated and replaced with 200 µl of EMEM containing DMSO, 50 or 100 µM LXR 623, or GSK 2033 at the same concentrations. From 1 through to 4 days post infection, 10 µl of 3-(4,5-dimethylthiazol-2-yl)-2,5-diphenyltetrazolium bromide (MTT) was added and incubated for 3 h. The media was aspirated and 100 of DMSO was added and incubated for 2 h followed by measuring the absorbance at 570 nm using a SpectraMax Plus 384 spectrophotometer (Molecular Devices). The assay was performed in triplicate with 5 technical replicates for each time point.

### Western blot analyses

Cells were washed twice with PBS and trypsinized. The cell pellets were washed twice in PBS followed by lysis with RIPA buffer (Abcam), containing a protease inhibitor cocktail (ThermoFisher) on ice for 30 min. The lysate was centrifuged at 12,000*g* for 10 min and supernatant were placed into clean tubes. Total protein was quantified using the BCA assay and 50 µg of sample were loaded onto PAGE gels and electrophoresed at 80 volts for 2 h. Protein was transferred onto PVDF membranes using an iBlot apparatus (Invitrogen) for 7 min. The membranes were blocked with Odyssey TBS Blocking Buffer (Licor) for 30 min at room temperature with shaking. Primary antibodies against ABCA1 (Sigma-Aldrich), LXR-α (Abcam) and ZIKV E (BioFront Technologies) were used at 1:1000 dilution, whereas anti-β-actin was diluted to 1:5000, and incubations were done at 4°C for 16 h. Antibody binding was detected with IRDye secondary antibodies (Licor) diluted to 1:5000 and the bands were visualized on an Odyssey CLx Imaging system (Licor).

### Analysis of inflammation gene expression using qPCR

SK-N-SH cells were seeded at 2 × 10^6^ cells/T75 flask and incubated at 37°C overnight. The cells were infected at a MOI of 10 for 1 h at 37°C with rocking. The cells were washed twice with PBS at 48 hpi and trypsinized. The trypsinized cells were washed twice with PBS and lysed in 1 ml TRIzol. Chloroform was added, and the preparation centrifuged at 4°C for 15 min. Total RNA in the aqueous phase was extracted using an RNAeasy kit according to manufacturer instruction. A microgram of total RNA was used to synthesize cDNA using a VILO cDNA synthesis kit (Invitrogen). The relative mRNA levels of *CXCL10* (assay IDHs00171042_m1), *RANTES/CCL5* (assay ID Hs00982282_m1), *IFN-1β* (assay ID Hs01077958_s1) and *TNF-α* (Hs00174128_m1) were determined from the cDNA on a QuantStudio 6 real time PCR system (ThermoFischer).

### Transmission electron microscopy

For transmission electron microscopy (TEM), samples were prepared and processed as described previously [13].

### Cholesterol efflux assay

SK-N-SH cells were seeded in a 6-well plate at 10^6^ cells/well and incubated overnight. Media was removed wells washed 3 times with PBS, and replaced with 2.5 ml serum-free DMEM containing DMSO, 50 or 100 μM LXR 623, 50 or 100 μM GSK 2033 and incubated for 6 h. After incubation, supernatants were collected, cell lysates prepared, and cholesterol quantitated using a colorimetric cholesterol quantitation kit (Sigma #MAK043).

### Statistical analyses

To determine if differences between data were significant, one way ANOVA was applied in GraphPad Prism. Significance between means was subsequently determined with multiple two-tailed *t*-tests.

## Results

### Activation of the LXR pathway restricts replication of the tick-borne Powassan virus (POWV)

**Figure 1.**
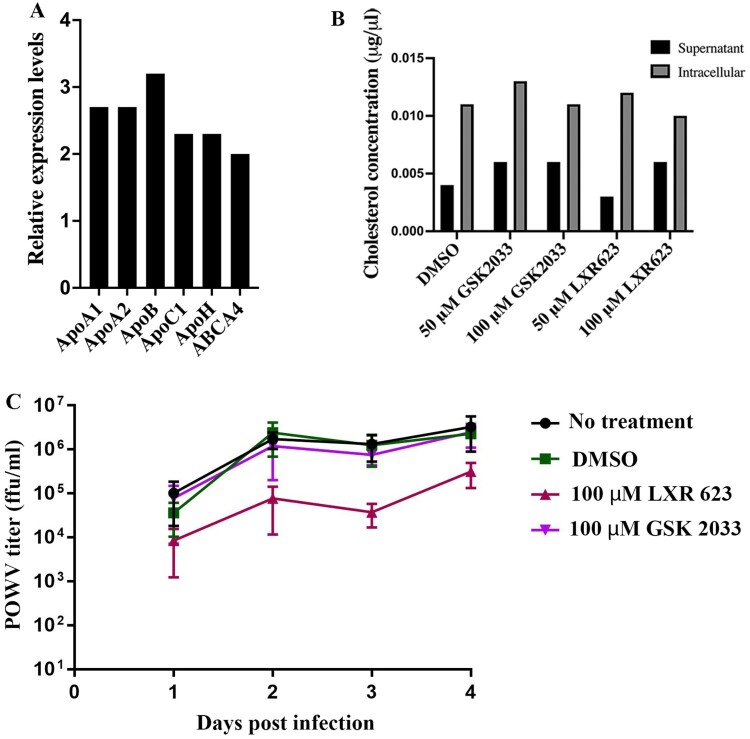
Analysis of the effect of LXR 623 on POWV replication. (A) Results from RNASeq suggesting that the LXR pathway is upregulated in *Peromyscus leucopus* mouse brains infected with POWV. The graph was drawn from the original dataset described in Mlera et al. [18] and the mRNA expression levels were in comparison to mock-infected brains. (B) Evaluation of cholesterol efflux from SK-N-SH cells treated with LXR 623. (C) SK-N-SH cells were infected with POWV at a MOI of 0.01 in the presence of the indicated compounds and supernatants collected at different time points for titration by immunofocus assay. The values represent means from 3 biological replicates and the error bars represent SD. ns, not significant; **, *p* < 0.01 (multiple *t*-tests).

Infection by the TBFV POWV in a reservoir host, *Peromyscus leucopus*, was not accompanied by the severe fatal neurological disease seen in laboratory mouse strains, but a rather mild inflammatory response and suppressed virus replication [21]. Transcriptomic analyses of the brain from these animals indicated upregulation of several apolipoproteins and ABCA4 (Figure 1(A)) suggesting late activation of the LXR pathway when POWV replication had declined [18]. Therefore, we investigated the effect of an LXR agonist LXR 623 on replication of the TBFV POWV in a neuroblastoma cell line SK-N-SH over a time-course. LXR 623 is a partial LXR-α and a full LXR-β agonist, which activates the ATP-binding cassette transporters (ABCA1 and ABCG1) leading to efflux of cholesterol out of the cell (Figure 1(B)). At a concentration of 100 µM, LXR 623 negatively impacted POWV replication by >10-fold compared to DMSO-treated cells (Figure 1(C)). Thus, LXR 623 mediated a disruption in POWV replication.

We also infected SK-N-SH cells with POWV in the presence of the LXR antagonist GSK 2033 and showed that it had no effect on POWV replication.

### Activation of the LXR pathway restricts replication of the mosquito borne Zika virus (ZIKV)

**Figure 2.**
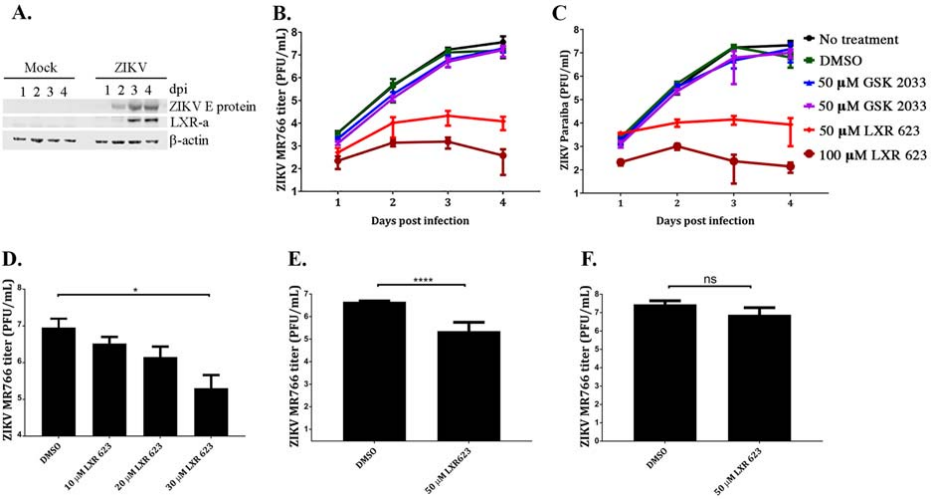
ZIKV infection upregulates LXR-*α* late in infection and LXR 623 negatively impacts ZIKV replication. (A) SK-N_SH cells were mock-infected or infected with ZIKV at a MOI of 1 and cell lysates collected over a 4-day time course. Western blot analysis was performed to detect changes in LXR-*α* expression. (B). Cells were seeded at 2 × 10^6^ cells/T75 flask, incubated overnight and infected at a MOI of 0.01 the next day. Infected cells were incubated with 50 µM LXR 623 for 48 h at 37°C and 5% CO_2_. Uninfected controls were incubated in EMEM. Replication kinetics of ZIKV MR766 in cells incubated in EMEM, DMSO, 50 or 100 µM LXR 623, or 50 or 100 µM GSK 2033 are shown. (C) Replication kinetics of ZIKV Paraiba in cells incubated in EME, DMSO, 50 or 100 µM LXR 623, or 50 or 100 µM GSK 2033. (D) Analysis of the effect of LXR 623 treatment at 10, 20 or 30 µM LXR 623. *p < 0.05. Evaluation of the impact of LXR 623 added to infected cell culture post infection. Infections were done at a MOI of 0.01 (E) or a high MOI of 10 (F). The inoculum was incubated with cells for 1 h followed by washing twice with PBS and replacement with drug-containing media. Values represent means from 3 biological replicates and error bars indicate SD from the mean. ****p < 0.0001; ns, not significant (multiple *t*-tests).

The recent emergence of the MBFV ZIKV in Latin American countries, with consequences that included microcephaly in neonates who contracted the infection *in utero,* was profound. For ZIKV, we started by asking if the virus had any effect on the LXR pathway and western blot analyses established that ZIKV increased LXR-α protein expression starting at 2 days post infection (dpi) with peak expression at 3 and 4 dpi (Figure 2(A)). The 3 dpi time point coincides with peak ZIKV titers in SK-N-SH cells [13]. Thus, we also evaluated the effect of activating the LXR pathway with LXR 623 on ZIKV replication in the neuroblastoma cell line SK-N-SH. To do this, we pretreated SK-N-SH cells with 50 or 100 µM LXR 623 and evaluated virus reproduction (ZIKV Paraiba or ZIKV MR766) over 4 dpi. At 2 dpi, levels of both ZIKV strains titers in cells treated with 100 µM LXR were a log less than titers from cells that were either not treated, treated with DMSO, or treated with the LXR antagonist GSK 2033 (Figure 2(B, C)). By 3 dpi, virus growth was reduced by 3 logs compared to controls, and virus replication remained reduced at 4 dpi. When treatment was with 50 µM LXR 623, the reduction of ZIKV MR766 titers was still reduced, but not as marked as with the 100 µM LXR 623 concentration (Figure 2(B)). Similar results were noted with ZIKV Paraiba (Figure 2(C)). These results demonstrated that growth of a MBFV was reduced when LXR was activated in SK-N-SH cells by LXR 623 treatment. The suppression of ZIKV replication by LXR 623 was more pronounced than POWV.

We also evaluated the effect of lower concentrations of LXR 623 on ZIKV replication using the MR766 strain for these experiments. Virus titration was performed at 3 dpi, and a dose-dependent inhibition of ZIKV MR766 replication was observed (Figure 2(D)). Thus, LXR 623 inhibition of ZIKV replication was dose-dependent and concentrations >20 µM LXR 623 were more effective.

Next, we determined how ZIKV replication would be affected if the compound was added when infection was already established. SK-N-SH cells were infected first, and then incubated with media containing LXR 623. These experiments were done at either a low multiplicity of infection (MOI) of 0.01 or a high MOI of 10 and virus was quantified by plaque assay at 3 dpi or 2 dpi, respectively. Compared to DMSO controls, SK-N-SH cells produced 100-fold less virus when infected at a MOI of 0.01 and then LXR 623 was added post infection (Figure 2(E)). However, 2.8-fold reduction in ZIKV titers was observed when the infecting MOI was high (Figure 2(F)). This result suggested that LXR 623 effects on suppressing ZIKV replication could still be realized post infection in a manner that was dependent on viral load.

### Activation of the LXR pathway inhibits endoplasmic reticulum expansion and biogenesis of flavivirus-induced vesicles

**Figure 3.**
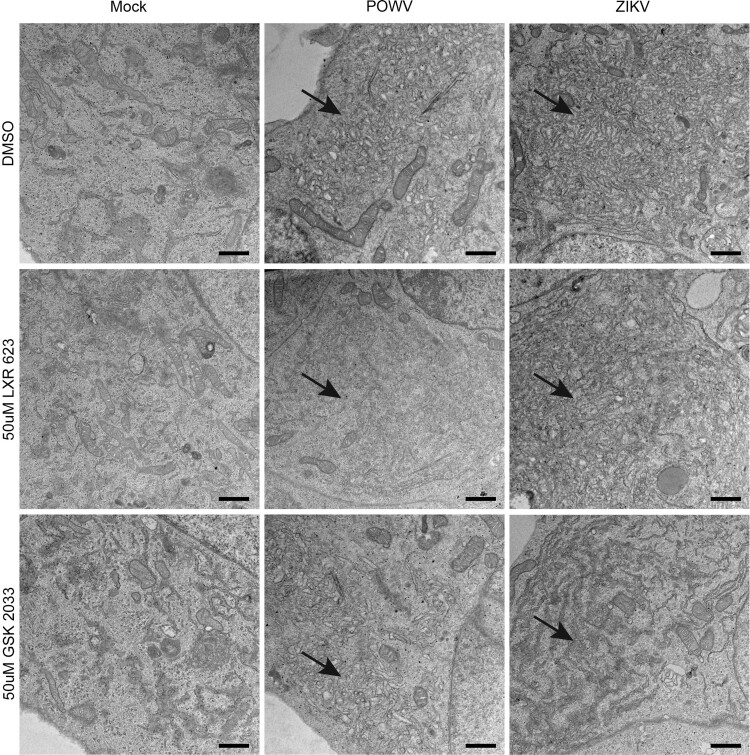
TEM of SK-N-SH cells infected with ZIKV in the presence or absence of LXR 623. SK-N-SH cells were seeded at 1 × 10^5^ cells/well on Thermanox coverslips in EMEM, DMSO, 50 uM LXR 623, or 50 uM GSK 2033. After 24 h treatment, cells were mock or virus infected (POWV LB or ZIKV MR766) at a MOI of 10, fixed, resin embedded, 70 nm section cut, and processed for TEM. The arrows point to ER expansion caused by flavivirus infection and the DMSO control represent the “classical” expansion. LXR 623 treatment in POWV infection led to failed ER expansion, but ZIKV induced some distorted expansion. Typical ER expansion was observed in POWV infection exposed to GSK 2033 and distorted membranes were also observed in ZIKV infection. Scale bar = 800 nm.

Flavivirus infection is associated with extensive expansion of ER-derived membranes and the generation of membrane-bound vesicles that encase viral replicative complexes (RCs) [11, 13, 15, 22]. The ER expansion and vesicle biogenesis require cholesterol and other lipid moieties. Thus, we employed transmission electron microscopy (TEM) to assess ER expansion and the generation of flavivirus-induced vesicles in LXR 623-treated cells. At 3 dpi, TEM results showed that flavivirus-infected cells treated with DMSO had extensive ER expansions and vesicle formation as expected (Figure 3). These membrane expansions were also observed in cells that were treated with the LXR antagonist GSK 2033.

In contrast, flavivirus-infected cells that were treated with 50 µM LXR 623, lacked ER expansions or vesicle formation (Figure 3). In the presence of LXR 623, ZIKV MR766-infected cells appeared to have expanded the ER or generated vesicles, but the architecture of these structures was aberrant in that their appearance was not consistent with how they looked in our previous studies [11, 13]. Combined, these results suggested that the mechanism of inhibition of flavivirus replication in the presence of LXR 623 was partly associated with disruption of membrane-bound vesicles and probable reduction of the ER platform for virus replication. Since these structures are obligatory for virus replication, this result was not surprising.

**Figure 4.**
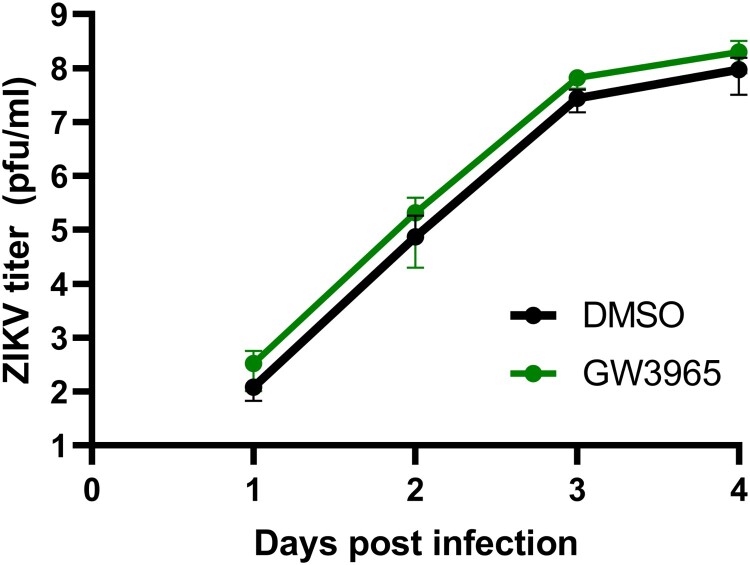
The LXR agonist GW3965 does not affect ZIKV replication. SK-N-SH cells were treated with 2 μM GW3965 then infected as described in Figure 2.

To investigate this further, we used a different LXR agonist to determine if the suppression of ZIKV was solely dependent on cholesterol efflux, which would lead to restricted formation of ER-derived vesicles. Surprisingly, exposure of SK-N-SH cells to the potent LXR agonist GW3965 did not result in notable differences in ZIKV replication when compared to DMSO-treated cells (Figure 4). This result was intriguing and suggested that the mechanism by which LXR 623 may be affecting flavivirus replication in SK-N-SH cells was different and not entirely dependent on cholesterol efflux.

### Activation of the LXR pathway induces higher antiviral cytokine expression in ZIKV infected cells

**Figure 5.**
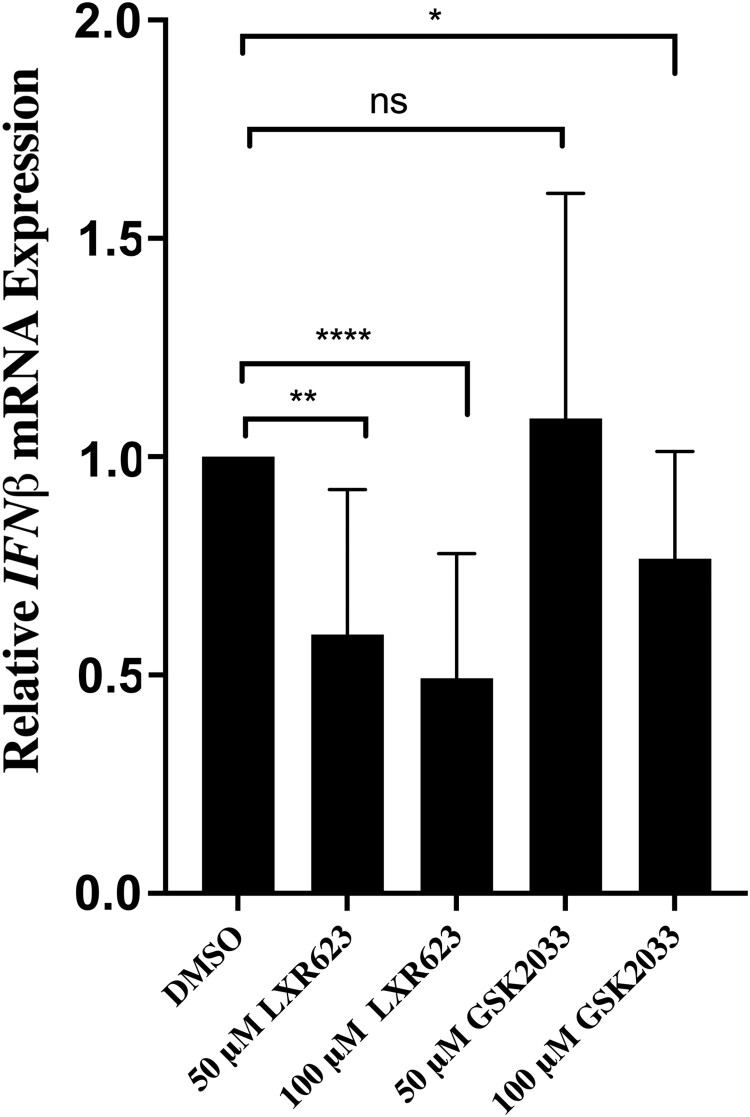
LXR 623 downregulates *IFN-β* transcripts. Uninfected SK-N-SH cells were treated with DMSO or LXR 623 at 50 μM. Cells were harvested at 24 h post treatment and total RNA was extracted. cDNA was synthesized and subjected to qPCR. *IFN-β* transcript expression levels were normalized to *HPRT* transcripts. Values were from 3 biological replicates and error bars represent SD. ***, *p* < 0.001; ****, *p* < 0.0001; ns, not significant (two tailed *t*-test).

ZIKV evades the innate immune response by inducing inflammation [23, 24]. In contrast, the LXR system suppresses inflammation by a unique mechanism known as transrepression [24, 25]. Trans-repression involves protein–protein interactions between nuclear receptors and promoter-bound transcription factors without direct sequence-dependent DNA interactions. To verify the anti-inflammatory effect of an activated LXR pathway, we used qPCR to measure the expression levels of transcripts encoding for the pro-inflammatory cytokine interferon β (IFN-β1). The results indicated that activation of LXR in SK-N-SH cells with LXR 623 in uninfected cells led to significantly lower *IFN-β* transcripts compared to DMSO treated cells (Figure 5). At 50 μM, GSK2033 did not affect *IFN-β* transcript levels as expected, but the higher concentration of 100 μM resulted in a slight decrease in *IFN-β* transcripts (Figure 5). Thus, LXR activation by LXR 623 was consistent with an anti-inflammatory response in uninfected cells.

**Figure 6.**
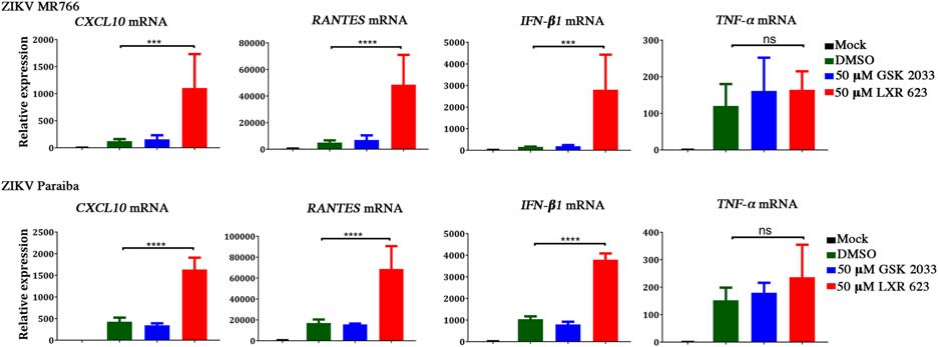
Effect of LXR 623 on the expression levels of cytokine-encoding mRNAs in ZIKV-infected SK-N-SH cells. Cells were infected at a MOI of 10, and transcript expression levels were normalized to GAPDH. Values represent the mean from of 3 biological replicates and error bars indicate SD from the mean. ***p < 0.001; ****p < 0.0001; ns, not significant.

In order to investigate a possible role for the LXR system in modulating the inflammatory cytokine response to ZIKV, we treated infected SK-N-SH cells with LXR 623 and used qPCR to determine the mRNA expression levels of 4 genes associated with inflammatory or antiviral responses, i.e. *CXCL10*, *RANTES*/*CCL5*, *IFN-β1* and *TNF-α*. We were intrigued to observe that infection of SK-N-SH cells with both ZIKV strains in the presence of 50 µM LXR 623 resulted in significantly higher mRNA expression levels for *CXCL10*, *CCL5* / *RANTES* and *IFN-β1* (Figure 6). Interestingly, *TNF-α* mRNA was not significantly affected by LXR 623. In contrast, treating ZIKV infected cells with either DMSO or, GSK 2033 had no effect on the mRNA expression levels of these cytokines (Figure 6). These results indicate that ZIKV infection negates the inflammation-suppressive effects of LXR 623, and that the drug itself may augment the virus-mediated induction of cytokines. Additional studies will be required to elucidate the mechanism.

### Activation of the LXR pathway protects SK-N-SH cells from ZIKV-induced cytopathic effects, but slows cell proliferation

**Figure 7.**
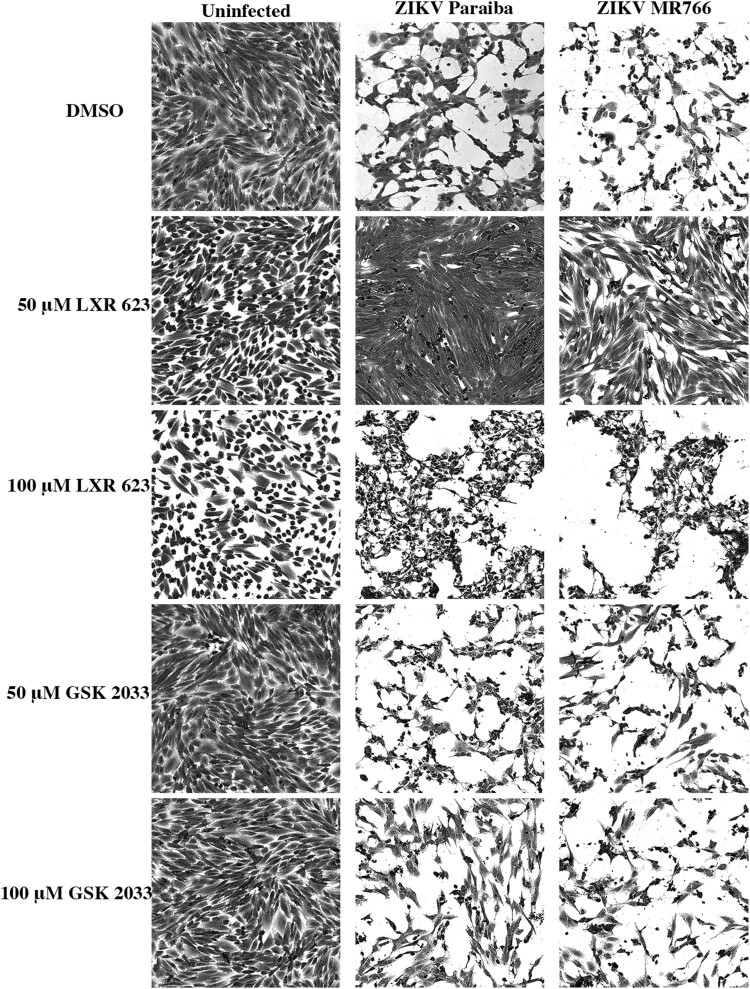
Protective effect of LXR 623 on ZIKV-infected SK-N-SH cells from apoptosis. Cells were pre-treated with DMSO, GSK2033 or LXR 623 followed by infection at a MOI of 0.01 and allowed to grow for 4 dpi. At 4 dpi, culture media was aspirated, and cells washed in PBS followed by fixing with 4% paraformaldehyde for 10 min. The fixative was removed, and cells were rinsed in PBS 3 times and then stained with Coomassie Brilliant Blue for 2 min. The stain was removed followed by 3 washes in PBS. Stained cells were imaged using a 40× objective on an AxioVert.A1 microscope (Zeiss) equipped with an Axiocam 503 mono camera.

The cytopathology of ZIKV is mediated via the activation of programmed cell death, and expression of cytokines, such as CXCL10 can contribute towards the induction of apoptosis [13, 26–28]. Infection of SK-N-SH cells in the presence of DMSO or 50 or 100 µM GSK 2033 produced extensive cytopathic effects (CPE) (Figure 7). However, treatment of infected cells with 50 µM LXR 623 greatly reduced cell death by 5 dpi (Figure 7). The protective effect of LXR 623 was dose-dependent because SK-N-SH cells exposed to 100 µM LXR 623 showed that cytotoxicity was enhanced by ZIKV infection (Figure 7). This conclusion was based on the observation that the100 µM LXR 623-treated monolayers appeared more intact than those that had the drug treatment and virus infection.

**Figure 8.**
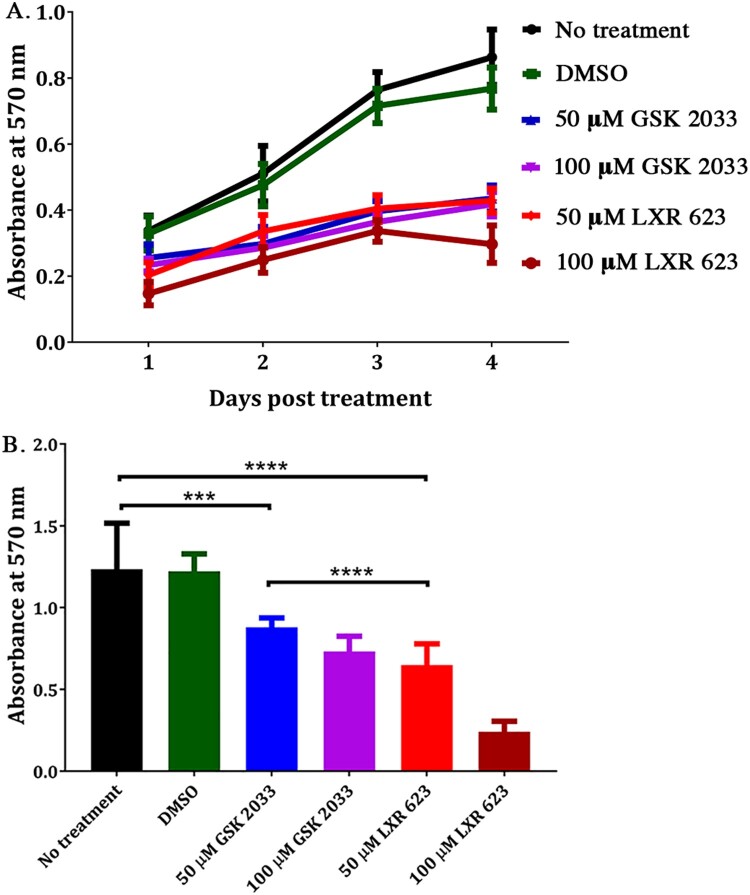
Proliferation of SK-N-SH cells in LXR 623. (A) SK-N-SH cells were seeded at 10^4^ cells/well. Proliferation was evaluated over the course of 4 days. (B) Cells were seeded at 5 × 10^4^ cells/well and the assay was done at 72 h post treatment. Values represent the mean from of 3 biological replicates and error bars indicate SD from the mean. ***p < 0.001; ****p < 0.0001 (multiple *t*-test).

Another LXR agonist, T0901317, is reported to negatively affect the proliferation of prostate and breast cancer cell lines *in vitro* [29]. We, therefore, evaluated the effect of LXR 623 on the proliferation of SK-N-SH cells at 50 or 100 µM concentrations and compared the results to untreated or DMSO-treated cells. The evaluation was done in SK-N-SH cells that were seeded at 10^4^ cells/well or 5 × 10^4^ cells/well in a 96-well plate. Figure 8(A) shows that treatment with LXR 623 or GSK 2033 significantly slowed the rate of proliferation of SK-N-SH cells as measured by the MTT assay. At 4 days post treatment (dpt), the rate of proliferation of SK-N-SH cells seeded at 10^4^ cells/well in 50 µM LXR 623 was 44% of the untreated cells. The rate of proliferation was the same at 4 dpt for cells in 50 or 100 µM GSK 2033.

The rate of proliferation for SK-N-SH cells seeded at 5 × 10^4^ cells/well and treated with 50 µM LXR 623 or 100 µM GSK 2033 was close to 60% of untreated cells (Figure 8(B)), suggesting that the rate of proliferation of cells in the compounds was also dependent on cell density. We also note that despite the slowed rate of proliferation, ZIKV-infected cells in 50 µM LXR 623 were protected from cell death (Figure 8). Therefore, LXR 623 appears to slow down cell proliferation and this reduced rate may also imply that fewer virus particles can be generated when compared to normally proliferating infected cells.

## Discussion

The role of lipid metabolism in flavivirus replication is now well-established [15, 17, 30, 31]. Cholesterol and other lipid moieties are required for ER expansion and biogenesis of membrane-bound vesicles, subcellular replication compartments in which virus replication occurs. In addition, flavivirus infection leads to an increase in cholesterol levels in the cell by boosting 3-hydroxy-3-methylglutaryl-coenzyme a reductase (HMGCOR), a rate limiting enzyme in cholesterol synthesis [32–34]. Thus, flavivirus reproduction depends on these cellular organelles, and several studies have demonstrated that disruption of lipid metabolism affects replication of flavivirus, such as dengue and West Nile virus [17, 35, 36]. Those studies targeted mostly the actual enzymes involved in lipid/cholesterol metabolism, such as HMGCoR. We reported that POWV infection in a reservoir host was associated with an upregulation of LXR, which is a critical regulator of lipid homeostasis [18]. Therefore, we sought to determine if targeting LXR would impair flavivirus replication. We examined both a TBFV and a MBFV, but primarily focused on ZIKA virus in this study.

ZIKV replicates to high titers in SK-N-SH cells, as well as in other neuroblastoma cell lines, and the infection leads to cell death [13, 37, 38]. The SK-N-SH is a neuroblastoma cell line originally developed from a bone marrow biopsy from a 4-year-old female and can be differentiated with retinoic acid to a neuronal lineage with extensive neurite growth [39, 40]. The brain contains approximately 20% of the body cholesterol and this increases the propensity of neuronal cells, such as the neuroblastoma SK-N-SH cell line, to be wired for highly controlled lipid metabolism through the LXR axis.

We demonstrated that permissive ZIKV replication in SK-N-SH cells was significantly reduced (*p* < 0.0001) if the infected cells were exposed the LXR agonist LXR 623. The inhibitory effect of LXR 623 was also observed with POWV, a TBFV. Our original hypothesis was that potentiation of the LXR pathway would decrease available intracellular cholesterol and, thus, would restrict virus replication by limiting ER expansion and membrane-bound vesicle biogenesis. TEM confirmed that these subcellular structures were absent or aberrant in the presence of LXR 623 (Figure 3). However, when we used a different small molecule compound GW3965, which is a potent LXR agonist, we observed that the drug had no effect on ZIKV replication. This observation suggests that cholesterol efflux may not be the critical mechanism by which LXR 623 inhibited flavivirus replication. Interference with viral proteins and/or RNA may be possible, and this requires further study.

ZIKV and other flavivirus infections trigger the expression of numerous cytokines of the innate immune response aimed at curtailing the viral invasion. We noted a strain dependent differences where the clinical isolate ZIKV Paraiba induced higher cytokine levels compared to the lab-adapted strain ZIKV MR766 (Figure 6). Thus, since MR766 has been passaged numerous times *in vitro*, this strain-dependent difference in cytokine expression suggested that adaptation of ZIKV MR766 to cell culture may have led to a reduced ability to induce higher cytokine levels. Interferons (IFNs) play a crucial role in the induction of some of these cytokines, such as CXCL10, as well as controlling viral infections [41–44]. The same may also aggravate pathogenesis, and thus the strain-dependent differences in the induction of cytokines may contribute towards differences in severity of clinical disease.

On the other hand, the LXR system is known to modulate metabolism, inflammation as well as innate and adaptive immune responses [45–48]. Regarding innate immune responses, LXRs reduce inflammation *in vivo*, and *in vitro* in colonic cells, leukocytes, macrophages and many other mouse cells by inhibiting IFN-γ-induced responses, modulation of IL-18 among others [49, 50]. However, a reduction in inflammation in the infected host may compromise immune responses aimed at the pathogen [45]. We noted that LXR activation with LXR 623 significantly enhanced the mRNA expression levels of *IFN-1β*, *CXCL10*, and *RANTES*/*CCL5* by several orders of magnitude, but not *TNF-α* (Figure 6). This was a surprising observation especially because there was no cell death associated with such a massive increase in cytokine mRNA expression (Figure 6). Thus, we hypothesize that the increase in these cytokines may be part of the mechanism by which LXR 623 inhibited ZIKV replication, in addition to depleting virus-induced vesicles and preventing ER expansions. However, we are uncertain if other LXR agonists not tested in our experiments would achieve the same results.

Inhibition of the LXR pathway system in the context of flavivirus infections has led to complex results. Neoechinulin B (NeoB) is an extract from the fungus *Aspergillus amstelodamii* that inhibits LXRs. A recent report demonstrated that inactivation of LXR by NeoB inhibited hepatitis C virus (HCV) replication by disrupting double-membrane vesicles believed to be HCV replication sites [51] and also suppressed poliovirus replication. However it was ineffective at inhibiting dengue virus [51]. In our study, we used GSK 2033 as an LXR antagonist which leads to lower expression of ABCA1 and observed that the vesicles were not disrupted during POWV infection, but the ultrastructure in ZIKV-infected cells was different from DMSO controls (Figure 3). In addition, GSK 2033 did not inhibit ZIKV infection, even at 100 µM. The mechanisms by which NeoB and GSK 2033 inhibit LXR may be different, and it might be interesting to establish the effect of NeoB on ZIKV or POWV replication. However, NeoB is currently not commercially available, and we were unable to include it our studies. At any rate, these findings suggested that the interface of the LXR system with flavivirus replication is complicated.

Lowering cholesterol levels is also a major aim in efforts to control coronary artery disease. Statins are licensed therapeutics for lowering cholesterol by inhibiting 3-hydroxy-methylglutaryl coenzyme A (HMG-CoA) reductase [52], and their role in inhibiting flavivirus replication has been explored. Lovastatin was shown to reduce dengue virus production by interfering with virion assembly in vitro, and it delayed infection as well as improved survival in AG129 mice, which are type II IFN deficient [53, 54]. However, lovastatin failed to inhibit dengue virus infection in a human clinical trial, and this could be because of partial and reversible reduction in cellular cholesterol [32]. In addition, type I IFN- responses are reported to negate the antiviral effects of statins on gamma herpesvirus infection [55], and this may also be the reason why lovastatin failed to be useful in dengue virus infection in humans. A synergistic effect on atherosclerosis and arterial plaque reduction was reported when LXR 623 was combined with the simvastatin [56]. It remains to be determined whether such an approach could change the outcome of dengue or other flavivirus infections.

Our observation that ZIKV infection in the presence of 50 µM LXR 623 did not induce cell death was intriguing (Figure 7). In addition, 50 µM LXR 623 enhanced cytokine expression in ZIKV-infected cells, which would be expected to increase cell death. These results were interesting because ZIKV pathogenesis is associated with virus-induced apoptosis [57–59]. A recent study also indicated that LXR 623 inhibits Chikungunya virus (CHIKV), which also depends on cholesterol metabolism for its replication [60]. In that study, the authors observed that infected cells were resistant to CHIKV-induced cytopathic effects partly because of upregulated IFN-stimulated gene ISG15 and general innate immune signaling [60]. Thus, activation of LXR during flavivirus infection *in vivo* may reduce cytopathology and inflammation to lower levels synergistic with increase cytokine expression.

We conclude that the LXR agonist LXR 623 significantly inhibits flavivirus replication in cell cultures partially by preventing the formation of vesicles and expansion of the ER. The treatment of SK-N-SH cells with LXR 623 also resulted in increased expression of cytokines that may play a role in establishing an antiviral state in the cells. The upregulation of cytokines by LXR 623 may be the key mechanism by which the drug inhibits flavivirus replication. In addition to increased cytokines, LXR 623 treatment also protects infected cells from apoptosis in a dose-dependent manner. Although LXR 623 reportedly causes untoward symptoms in healthy individuals, other LXR agonists should be explored for activity against flaviviruses. Thus, the LXRs are a lucrative target for inhibiting flavivirus replication, and the knowledge presented here should be applied in the rational design of therapeutics for these viruses.
